# Development of the Nordic Nutrition Recommendations 2023 Food-Based Diet Score and Its Association with All-Cause Mortality in Two Swedish Cohorts

**DOI:** 10.1016/j.tjnut.2025.06.030

**Published:** 2025-07-02

**Authors:** Anne B Mørch, Daniel B Ibsen, Alicja Wolk, Christina C Dahm

**Affiliations:** 1 Department of Public Health, Aarhus University, Aarhus, Denmark; 2 Department of Environmental Medicine, Karolinska Institutet, Stockholm, Sweden; 3 Department of Nutrition, Exercise and Sports, University of Copenhagen, Copenhagen, Denmark; 4 Steno Diabetes Center Aarhus, Aarhus University Hospital, Aarhus, Denmark

**Keywords:** Cohort study, sustainable diet, food-based dietary guidelines, diet score, mortality

## Abstract

**Background:**

The 2023 Nordic Nutrition Recommendations (NNR23) presented a dual focus on disease prevention and planetary health.

**Objectives:**

We aimed to develop a food-based diet score measuring adherence to NNR23 and assess its association with all-cause mortality in a Swedish population.

**Methods:**

We developed a novel diet score with 15 food components representing NNR23. Each component was scored between 0 and 1 point on a continuous scale, 1 being full adherence, based on participants’ intakes. The study population included women (48–83 y old) from the Swedish Mammography Cohort (*n =* 39,984) and men (45–79 y old) from the Cohort of Swedish Men (*n =* 48,850), who completed food frequency questionnaires in 1997, 2009, and 2019 and were followed up although linkage to the National Death Register. Multivariable adjusted Cox proportional hazards regression models with age as the underlying timescale were used to estimate hazard ratios (HRs) with 95% confidence intervals (CIs) for the association between adherence to NNR23 and all-cause mortality, as well as cardiovascular- and cancer-specific deaths.

**Results:**

The median NNR23 score was 9.5 (p10, p90; 8.3, 11) for women and 8.9 (p10, p90; 7.4, 10) for men; no participant achieved full adherence. During a median 18.8 y of follow-up, 30,142 participants died. Participants with the highest adherence (>10 points) at baseline had a 23% lower all-cause mortality (HR 0.77; 95% CI: 0.74, 0.80) compared with the lowest adherence group (<8 points). Results were similar for cardiovascular- and cancer-specific mortality. For long-term average food intake, we found an even lower mortality risk when comparing the highest adherence with the lowest adherence (HR 0.38; 95% CI: 0.37, 0.40).

**Conclusions:**

With a new food-based diet score representing NNR23, we found that higher compared with lower adherence to NNR23 was associated with lower mortality in a Swedish population.

## Background

The food we eat is a major contributory factor to mortality as suboptimal nutrition increases risk of noncommunicable diseases, ultimately leading to premature mortality [[1], [2], [3]]. The current global food system also causes 40% of global greenhouse gas emissions and contributes to the depletion of the planet’s resources [4,5]. The updated Nordic Nutrition Recommendations 2023 (NNR23) consider both human and planetary health, emphasizing reducing meat consumption and increasing consumption of vegetables, fruits and berries, cereals, nuts, and pulses [6]. The food-based NNR23 takes the environmental impact of diet into account as measured by greenhouse gas emissions, land use, water use, and nitrogen and phosphorus utilization. The range and limits for each food component were adjusted to align with both health and environmental objectives [6]. A diet score has previously been developed based on NNR23 recommendations focusing on macro- and micronutrients [7]. However, there is currently no food-based diet score designed to capture the food-based dietary recommendations of NNR23.

Other dietary guidelines focusing on planetary boundaries, such as the EAT-Lancet reference diet—which advocates for minimal inclusion of foods of animal origin [5]—have shown associations between higher adherence and lower mortality [5,8]. Although the EAT-Lancet diet is meant to represent a universal ideal diet with strict thresholds on all animal products [9,10], NNR23 also considers regional, cultural and practical aspects of eating, aiming to balance health benefits with environmental sustainability in a way that is more adaptable to everyday dietary practices in the Nordic countries [5,6,9]. For instance, due to culturally diverse ways of preparing potatoes, the EAT-Lancet diet categorized potatoes as a food for limited intake, unlike NNR23, in which boiled or baked potatoes are recommended to be eaten as part of a healthy and environmentally friendly diet [6,9]. Whether the pragmatic NNR23 guidelines are associated with a lower risk of premature mortality remains unknown.

This study aimed to develop a food-based diet score to represent adherence to NNR23 and investigate the association between adherence to the NNR23 food-based diet score and risk of all-cause mortality, as well as cardiovascular- and cancer-specific mortality.

## Methods

### Study population

This study was based on dietary data from women aged 48–83 from the Swedish Mammography Cohort (SMC, *n =* 39,984) and men aged 45–79 from the Cohort of Swedish Men (COSM, *n =* 48,850). All women living in Uppsala or Västmanland counties and born between 1914 and 1948 were invited into the SMC during 1987–1990 (response rate 72%). In late 1997, a questionnaire on diet, lifestyle, anthropometrics, and sociodemographics was sent to the women still alive and living in the study area. At the same time, all men born 1918–1952 living in Västmanland and Örebro counties were invited into COSM using the same questionnaire as in the SMC. In total, 48,850 men (49% of the invited) and 39,984 women (70% of the invited) participated in the 1997 assessment. Additional follow-up questionnaires on diet and lifestyle were sent in 2009 and 2019 to all participants still alive and who had completed the FFQ in 1997. In 2009, responses were received from 26,100 men (90%) and 25,259 women (84%), and in 2019, 16,166 men (86%) and 14,923 women (79%) [11,12]. For this study, the 1997 data collection was regarded as the baseline. We excluded participants with missing or incorrect identification numbers, who had died or had a cancer or cardiovascular disease diagnosis before 1 January 1998. Participants with implausible total energy intakes (±3 SD of mean total energy intake on the log scale) or missing information on dietary factors were also excluded. A total of 76,122 participants (41,118 men and 35,004 women) were included in the analyses (Supplemental Figure 1).

### Diet assessment

In late 1997, participants were asked to fill out a 96-item food frequency questionnaire (FFQ-1997) regarding the average consumption of food, drinks, and meals during the past 12 months. Response categories in the FFQ ranged from “never” to “>3 times a day” [12]. The study complies with the Declaration of Helsinki and was approved by the Regional Ethical Review Board at Karolinska Institute, Stockholm, Sweden. The 2 population-based cohorts (COSM and SMC) were registered at clinicaltrials.gov with identifiers NCT01127711 and NCT01127698, respectively, and both cohorts belong to the National Research Infrastructure SIMPLER (Swedish Infrastructure for Medical Population-based Life-course and Environmental Research; www.simpler4health.se). Completion and return of the questionnaires were considered consent. The follow-up FFQ-2009 was extended to 149 items, and FFQ-2019 contained 148 items (a question about sardine intake was not included) [13]. Average intakes of food were calculated based on age-specific portion sizes obtained from weighed diet records in age subgroups of the cohorts’ participants. An average daily energy intake was estimated using the Swedish Food Administration Database [14]. The validity of the 1997 FFQ has been evaluated in a random sample of 129 women from SMC by comparing the FFQ-based food estimates with 4 1-wk weighed diet records over 1 y. The following correlation coefficients were observed: 0.5–0.7 for fruits, 0.4–0.6 for vegetables, 0.5–0.7 for whole grains, 0.4–0.6 for dairy, 0.3–0.7 for red and processed meats, and 0.6 for sweetened beverages [15].

#### Development of the NNR23 diet score

The NNR23 diet score included the food groups for which intake recommendations were made in the NNR23 report (Supplemental Table 1) [6]. These were vegetables, fruit, and berries, wholegrain cereal, pulses, nuts and seeds, unsaturated oils, fish and seafood, egg, potatoes, white meat, juice, milk and dairy, red meat, processed meat, added sugar, and caffeine derived from coffee and tea. Although caffeine is not a food as such, NNR23 does not define limits on cups of coffee or tea, but offers a limit of total caffeine intake, and therefore, we use this estimate [6]. We categorized food groups into “encourage,” “moderate,” and “discourage” consumption according to the health and environmental considerations in NNR23. Encouraged foods are considered to be positively associated with health and with low environmental impact. Foods to eat in moderation are weakly or not associated with negative health effects or moderately associated with climate impact. Discouraged foods are observed to have negative health effects and negative climate impact [6]. The threshold intake for full adherence for each food group was the recommended intake proposed in NNR23 guidelines (Supplemental Table 1, Supplemental Table 2). Food group recommendations were either stated as a specific amount or within a range. If a recommended range was proposed, the lower limit of the range was deemed sufficient for full adherence. Intakes twice the upper limit of the recommended intake were used as a cutoff for excessive consumption because NNR23 recommends dietary variety and keeping an appropriate energy balance [6]. NNR23 did not quantify a recommended intake for pulses, egg, potatoes, juice, and added sugar, and in such cases, we used referenced health benefits within certain intake ranges from the individual food chapters of the NNR23 report as a guide. For example, NNR23 does not quantify recommended intake of potatoes but refers to positive health effects at intakes in the range of 50–130 g/d, and we chose to adopt this range into the score (Supplemental Table 1) [6].

Food components were scored on a continuous scale from 0 to 1, where 0 points indicated no adherence and 1 point that the guideline was fully met. For components with minimum intake recommendation, scores were calculated as the ratio between the actual intake and the recommended intake (actual intake/recommended), and intakes exceeding the minimum recommended intake did not result in extra assigned points. For components with an upper limit of recommended intake, scores were calculated as 1 minus the difference between the actual intake and the recommended intake divided by the recommended intake [1 – (actual intake – recommended intake)/(upper limit intake − recommended intake)]. Participants consuming twice the maximum recommended intake received a score of 0 points. The guidelines concerning vegetables, fruits and berries, and fish intake consisted of a recommended lower limit of total intake and a recommended lower limit of specific intake of vegetables and of fatty fish, respectively. The scores for these 2 guidelines were constructed as a weighted sum of the subcomponents using equal weights and the score for the guideline on vegetables, fruit and berries was calculated as follows: [(total intake vegetables, fruit and berries/500) × 0.5] + [(intake vegetables/250) × 0.5] and for the fish component: [(total intake fish/350) × 0.5] + [(intake fatty fish/200) × 0.5].

#### Assessment of covariates

Potential confounders were selected based on relevant literature [[15], [16], [17]] and construction of a directed acyclic graph (Supplemental Figure 2) of the presumed relationship between adherence to the NNR23 diet score and mortality. Information on sex (men, women), educational level (primary, high school, university), sleep (h/d; < 6, 6–<7, 7–<8, 8–<9, >9), smoking status [never, former smokers (cigarettes/d; <20, 20–<40, or >40), or current smokers (cigarettes/d ; <20, 20–<40, or >40), alcohol intake (g/d), walking/cycling (min/d; <20, 20–<40, 40–<60, 60–<90, >90), dietary supplement use (regularly, sometimes, no), BMI (in kg/m^2^; <18.5, 18.5–<25, 25–<30, >30), derived from height and weight], hypertension (yes, no), hypercholesterolemia (yes, no), and diabetes (yes, no) was retrieved from the self-reported baseline questionnaires from 1997 [13,16]. Information on income in 1997 was retrieved through linkage to the longitudinal integrated database for health insurance and labor market studies and includes the entire Swedish population aged ≥ 16 y registered and alive on December 31 each year since 1990. Income data are reported as gross annual income based on reports from the Swedish tax authority [18]. Income was classified into quintiles ranging from <88,400 to >176,000 Swedish Kronor (SEK). Missing values in covariates were coded as separate missing categories.

### All-cause-, cancer-, and cardiovascular mortality ascertainment

Date of death was retrieved through linkage to the Death Register at Statistics Sweden using the Swedish personal identification number. All deaths are recorded in the register within 30 d [16]. Cause-specific mortality was ascertained for cardiovascular mortality (codes I00–I78) and cancer mortality (codes C00–C97) [19].

### Statistical analysis

Baseline characteristics of participants were presented by degree of adherence to the NNR23 food-based diet score, stratified by sex to identify sex-specific characteristics.

Cox proportional hazards regression models were used to estimate hazard ratios (HRs) with 95% confidence intervals (CIs) for the association between adherence to NNR23 and all-cause mortality, using age as the underlying timescale [20]. The assumption of proportional hazards was assessed using log–log plots, and no evidence for violation of the assumption was detected. The association was also investigated as a restricted cubic spline with 4 knots, with the reference point in the median (9.2 points). To test nonlinearity, we compared the restricted cubic spline model to a linear model using a likelihood ratio test. A statistically significant result (*P* < 0.05) suggests that the association deviates from linearity. [21].

To examine long-term adherence, dietary information was averaged with data from 2009 to 2019, when available. For participants with multiple dietary data points, we used the average intake across these intakes to represent their long-term average intake. Participants were followed from 1 January, 1998 until the date of death, or 31 December, 2019, whichever came first [12,15,17]. The main analysis was repeated for cardiovascular and cancer-specific mortality.

Model 1 was adjusted for age and sex (men, women) and total energy intake. Model 2 was further adjusted for educational level, income, sleep, smoking status, alcohol intake, physical activity, and dietary supplement use. Model 3 was additionally adjusted for BMI, and prevalence of hypertension, hypercholesterolemia, and diabetes at baseline. We consider model 3 our main model.

In a sensitivity analysis, we excluded 1 food component at a time from the NNR23 score to evaluate whether individual items might affect the overall results. The main analysis (model 3) was stratified in turn by sex, BMI, diabetes status, and income level to explore potential effect modification. Additionally, we assessed robustness by excluding deaths occurring within the first year of follow-up. Sensitivity analyses were also performed using diet score data from different time combinations (1997–2009, 1997–2019, and 1997–2009–2019).

All analyses were conducted using R (version 4.3.1; R Foundation for Statistical Computing) except for the restricted cubic spline analysis, which was conducted with Stata (StataCorp. 2023. Stata Statistical Software: Release 15. College Station, TX: StataCorp LLC.). For all analyses, a *P* value of 5% was considered significant. The data supporting this article are classified as sensitive under the European General Data Protection Regulation and cannot be publicly shared. However, access to the data and underlying code can be granted upon reasonable request through the Swedish National Research Infrastructure, SIMPLER (www.simpler4health.se).

## Results

At baseline, the median age of women in the study was 60 y (p10–p90: 51, 75) and of men 58 y (p10–p90: 48, 74). The NNR23 food-based score was 9.5 (p10–p90: 8.3, 11) and 8.9 (p10–p90: 7.4, 10) for women and men, respectively. None of the participants in the population achieved a full 15 points of adherence to the NNR23 score. Women with the highest adherence tended to be slightly older, have longer education, higher incomes, more adequate sleep, be more likely to be never-smokers, consume more alcohol, walk or cycle more frequently, take dietary supplements, have a normal weight, be less likely to have hypertension, have a higher energy intake, and follow an overall healthier diet compared with lowest adherence (Table 1). Men with the highest adherence shared many of these traits (Table 2). However, unlike women, men with the highest adherence had a lower energy intake compared with the lowest adherence.

**TABLE 1:** Characteristics of women (*N =* 35,004) in the study population stratified on NNR23 diet score at baseline.

	0–8 points (*n =* 2317)	>8–9 points (*n =* 7741)	>9–10 points (*n =* 14,293)	>10–13 points (*n =* 10,653)	Overall (*n =* 35,004)
NNR23 diet score (points), median (p10, p90)	7.6 (6.7, 7.9)	8.6 (8.2, 8.9)	9.5 (9.1, 9.9)	10 (10, 11)	9.5 (8.3, 11)
Age (y), median (p10, p90)	58 (50, 76)	59 (50, 75)	60 (51, 75)	61 (51, 75)	60 (51, 75)
Education					
Primary	1125 (49%)	3486 (45%)	5825 (41%)	3871 (36%)	14,307 (41%)
High School	888 (38%)	2957 (38%)	5737 (40%)	4309 (40%)	13,891 (40%)
University	285 (12%)	1259 (16%)	2675 (19%)	2415 (23%)	6634 (19%)
Missing	19 (1%)	39 (1%)	56 (0%)	58 (1%)	172 (0%)
Annual gross income, SEK					
< 88,400	796 (34%)	2608 (34%)	4754 (33%)	3592 (34%)	11,750 (34%)
88,400–114,000	582 (25%)	1795 (23%)	3145 (22%)	2234 (21%)	7756 (22%)
114,000–141,000	452 (20%)	1563 (20%)	2874 (20%)	2050 (19%)	6939 (20%)
141,000–176,000	302 (13%)	1076 (14%)	2143 (15%)	1629 (15%)	5150 (15%)
>176,000	184 (8%)	695 (9%)	1372 (10%)	1144 (11%)	3395 (10%)
Missing	< 5	< 5	5 (0.0%)	< 5	14 (0.0%)
Sleep (h/24 h)					
<6 h	164 (7%)	448 (6%)	755 (5%)	543 (5%)	1910 (5%)
6–7 h	405 (17%)	1447 (19%)	2543 (18%)	1961 (18%)	6356 (18%)
7–8 h	760 (33%)	2734 (35%)	5339 (37%)	3953 (37%)	12,786 (37%)
8–9 h	728 (31%)	2490 (32%)	4660 (33%)	3477 (33%)	11,355 (32%)
>9 h	203 (9%)	496 (6%)	795 (6%)	553 (5%)	2047 (6%)
Missing	57 (2.5%)	126 (1.6%)	201 (1.4%)	166 (1.6%)	550 (1.6%)
Smoking (cigarettes/d)					
Never	986 (43%)	3676 (47%)	7643 (53%)	6179 (58%)	18,484 (53%)
Former, <20	305 (13%)	1261 (16%)	2557 (18%)	1971 (19%)	6094 (17%)
Former, 20–<40	97 (4%)	293 (4%)	479 (3%)	316 (3%)	1185 (3%)
Former, ≥40	5 (0%)	21 (0%)	34 (0%)	24 (0%)	84 (0%)
Current, <20	355 (15%)	1129 (15%)	1782 (12%)	1165 (11%)	4431 (13%)
Current, 20–<40	387 (17%)	922 (12%)	1140 (8%)	585 (5%)	3034 (9%)
Current, ≥40	44 (2%)	90 (1%)	106 (1%)	41 (0%)	281 (1%)
Missing	138 (6%)	349 (5%)	552 (4%)	372 (3%)	1411 (4%)
Alcohol (g/d)	1.5 (0, 9.9)	2.2 (0, 11)	2.6 (0, 10)	2.7 (0, 10)	2.5 (0, 10)
Walking/cycling (min/d)					
Never/seldom	376 (16%)	961 (12%)	1289 (9%)	773 (7%)	3399 (10%)
<20	452 (20%)	1455 (19%)	2571 (18%)	1612 (15%)	6090 (17%)
20–<40	540 (23%)	2359 (30%)	4677 (33%)	3603 (34%)	11,179 (32%)
40–<60	308 (13%)	1128 (15%)	2500 (17%)	2101 (20%)	6037 (17%)
60–90	194 (8%)	654 (8%)	1339 (9%)	1137 (11%)	3324 (9%)
>90	146 (6%)	467 (6%)	907 (6%)	743 (7%)	2263 (6%)
Missing	301 (13.0%)	717 (9.3%)	1010 (7.1%)	684 (6.4%)	2712 (7.7%)
Dietary supplement use					
No	1100 (47%)	3682 (48%)	6096 (43%)	4056 (38%)	14,934 (43%)
Sometimes	517 (22%)	1859 (24%)	3934 (28%)	3199 (30%)	9509 (27%)
Regularly	460 (20%)	1602 (21%)	3283 (23%)	2695 (25%)	8040 (23%)
Missing	240 (10%)	598 (8%)	980 (7%)	703 (7%)	2521 (7%)
BMI (kg/m^2^)					
Underweight (BMI <18.5)	75 (3%)	139 (2%)	203 (1%)	130 (1%)	547 (2%)
Normal weight (BMI 18.5–<25)	1169 (50%)	4084 (53%)	7662 (54%)	5892 (55%)	18,807 (54%)
Overweight (BMI 25–<30)	729 (31%)	2515 (32%)	4707 (33%)	3513 (33%)	11,464 (33%)
Obese (BMI >30)	263 (11%)	860 (11%)	1489 (10%)	981 (9%)	3593 (10%)
Missing	81 (3.5%)	143 (1.8%)	232 (1.6%)	137 (1.3%)	593 (1.7%)
Hypertension					
Yes	440 (19%)	1544 (20%)	2806 (20%)	2268 (21%)	7058 (20%)
Diabetes					
Yes	74 (3%)	244 (3%)	486 (3%)	447 (4%)	1251 (4%)
Hypercholesterolemia					
Yes	158 (7%)	529 (7%)	1126 (8%)	882 (8%)	2695 (8%)
Dietary intake, median (p10, p90)					
Total energy (kcal/d)	1612 (959, 2779)	1593 (1041, 2436)	1668 (1155, 2359)	1752 (1253, 2374)	1680 (1160, 2402)
Fruits, berries, and vegetables (g/d)	210 (72, 650)	270 (120, 570)	330 (170, 620)	430 (240, 700)	340 (150, 650)
Whole grains (g/d)	14 (0, 150)	32 (0, 180)	64 (13, 180)	95 (32, 200)	64 (9.1, 180)
Pulses (g/d)	16 (0, 32)	16 (0, 51)	16 (0, 51)	32 (16, 67)	16 (0, 51)
Nuts (g/d)	0 (0, 1.3)	0 (0, 1.3)	0 (0, 1.3)	0 (0, 1.3)	0 (0, 1.3)
Unsaturated oil	0 (0, 0)	0 (0, 0)	0 (0, 0)	0 (0, 0)	0 (0, 0)
Total fish (g/d)	19 (4.4, 64)	21 (8.8, 44)	27 (12, 48)	36 (17, 57)	28 (12, 51)
Egg (g/d)	3.1 (0, 21)	3.1 (0, 19)	7.9 (2.5, 19)	7.9 (2.5, 19)	7.9 (2.5, 19)
Potatoes (g/d)	74 (9.0, 230)	74 (25, 140)	74 (34, 130)	78 (50, 120)	74 (32, 130)
White meat (g/d)	7.7 (0, 26)	8.2 (0, 25)	8.2 (0, 25)	8.2 (0, 26)	8.2 (0, 26)
Juice (g/d)	11 (0, 160)	11 (0, 160)	11 (0, 140)	11 (0, 85)	11 (0, 140)
Dairy products (g/d)	150 (0, 730)	220 (31, 750)	300 (110, 690)	350 (180, 600)	300 (84, 680)
Red meat (g/d)	35 (9.7, 110)	31 (11, 58)	30 (14, 53)	29 (9.7, 45)	30 (11, 53)
Processed meat (g/d)	28 (11, 76)	28 (9.7, 54)	27 (7.5, 53)	19 (2.7, 49)	25 (6.5, 53)
Added sugar (g/d)	10 (10, 20)	10 (10, 15)	10 (10, 12)	10 (10, 11)	10 (10, 13)
Caffeine (mg/d)	290 (86, 660)	260 (110, 490)	250 (120, 430)	240 (120, 400)	250 (110, 440)

Abbreviations: *N,* sample size; NNR23, Nordic Nutrition Recommendations 2023.

**TABLE 2:** Characteristics of men (*N =* 41,118) in the study population stratified on NNR23 diet score at baseline.

	0–8 points (*n =* 8909)	>8–9 points (*n =* 13,217)	>9–10 points (*n =* 13,301)	>10–13 points (*n =* 5691)	Overall (*n =* 41,118)
NNR23 diet score (points) median (p10, p90)	7.4 (6.5, 7.9)	8.6 (8.1, 8.9)	9.4 (9.1, 9.9)	10 (10, 11)	8.9 (7.4, 10)
Age (y) median (p10, p90)	55 (47, 71)	58 (47, 73)	60 (48, 74)	60 (49, 75)	58 (48, 74)
Education					
Primary	3362 (38%)	4703 (36%)	4208 (32%)	1551 (27%)	13,824 (34%)
High School	4284 (48%)	6327 (48%)	6708 (50%)	2831 (50%)	20,150 (49%)
University	1228 (14%)	2150 (16%)	2340 (18%)	1298 (23%)	7016 (17%)
Missing	35 (0%)	37 (0%)	45 (0%)	11 (0%)	128 (0%)
Annual gross income, SEK					
0–88,400	1030 (12%)	1115 (8%)	968 (7%)	403 (7%)	3.516 (9%)
88,400–114,000	1620 (18%)	2461 (19%)	2392 (18%)	960 (17%)	7433 (18%)
114,000–141,000	1869 (21%)	2684 (20%)	2682 (20%)	1052 (18%)	8287 (20%)
141,000–176,000	2204 (25%)	3262 (25%)	3239 (24%)	1349 (24%)	10,054 (24%)
>176,000	2181 (24%)	3691 (28%)	4017 (30%)	1923 (34%)	11,812 (29%)
Missing	5 (0.1%)	<5	<5	<5	16 (0.0%)
Sleep (h/24 h)					
<6 h	446 (5%)	502 (4%)	458 (3%)	186 (3%)	1592 (4%)
6–7 h	1762 (20%)	2458 (19%)	2307 (17%)	979 (17%)	7506 (18%)
7–8 h	3348 (38%)	5223 (40%)	5339 (40%)	2347 (41%)	16,257 (40%)
8–9 h	2659 (30%)	4137 (31%)	4285 (32%)	1819 (32%)	12,900 (31%)
> 9 h	560 (6%)	748 (6%)	754 (6%)	304 (5%)	2366 (6%)
Missing	134 (1.5%)	149 (1.1%)	158 (1.2%)	56 (1.0%)	497 (1.2%)
Smoking (cigarettes/d)					
Never	2709 (30%)	4723 (36%)	5254 (40%)	2340 (41%)	15,026 (37%)
Former, <20	1729 (19%)	2940 (22%)	3207 (24%)	1457 (26%)	9333 (23%)
Former, 20–<40	878 (10%)	1377 (10%)	1239 (9%)	494 (9%)	3988 (10%)
Former, ≥40	207 (2%)	245 (2%)	248 (2%)	103 (2%)	803 (2%)
Current, <20	922 (10%)	1245 (9%)	1163 (9%)	442 (8%)	3772 (9%)
Current, 20–<40	1362 (15%)	1314 (10%)	1045 (8%)	353 (6%)	4074 (10%)
Current, ≥40	557 (6%)	507 (4%)	343 (3%)	126 (2%)	1533 (4%)
Missing	545 (6%)	866 (7%)	802 (6%)	376 (7%)	2589 (6%)
Alcohol (g/d), median (p10, p90)	7.0 (0, 23)	7.9 (0.37, 23)	8.5 (0.61, 22)	9.2 (0.87, 23)	8.1 (0.38, 23)
Walking/cycling (min/d)					
Never/seldom	1446 (16%)	1652 (12%)	1338 (10%)	481 (8%)	4917 (12%)
<20	1950 (22%)	3088 (23%)	2886 (22%)	1214 (21%)	9138 (22%)
20–<40	2047 (23%)	3365 (25%)	3812 (29%)	1698 (30%)	10,922 (27%)
40–<60	984 (11%)	1800 (14%)	1964 (15%)	931 (16%)	5679 (14%)
60–90	595 (7%)	999 (8%)	1178 (9%)	507 (9%)	3279 (8%)
>90	765 (9%)	1054 (8%)	1013 (8%)	451 (8%)	3283 (8%)
Missing	1122 (12.6%)	1259 (9.5%)	1110 (8.3%)	409 (7.2%)	3900 (9.5%)
Dietary supplement use					
No	6081 (68%)	8544 (65%)	8204 (62%)	3191 (56%)	26,020 (63%)
Sometimes	1146 (13%)	2064 (16%)	2323 (17%)	1126 (20%)	6659 (16%)
Regularly	1004, (11%)	1706 (13%)	1947 (15%)	1017 (18%)	5674 (14%)
Missing	678 (8%)	903 (7%)	827 (6%)	357 (6%)	2765 (7%)
BMI (kg/m^2^)					
Underweight (BMI <18.5)	54 (1%)	58 (0%)	53 (0%)	16 (0%)	181 (0%)
Normal weight (BMI 18.5–<25)	3539 (40%)	5516 (42%)	5705 (43%)	2545 (45%)	17,305 (42%)
Overweight (BMI 25–<30)	3825 (43%)	5732 (43%)	5821 (44%)	2464 (43%)	17,842 (43%)
Obese (BMI >30)	981 (11%)	1223 (9%)	1135 (9%)	413 (7%)	3752 (9%)
Missing	510 (5.7%)	688 (5.2%)	587 (4.4%)	253 (4.4%)	2038 (5.0%)
Hypertension					
Yes	1674 (19%)	2699 (20%)	2905 (22%)	1289 (23%)	8567 (21%)
Diabetes					
Yes	466 (5%)	753 (6%)	761 (6%)	356 (6%)	2336 (6%)
Hypercholesterolemia					
Yes	968 (11%)	1491 (11%)	1570 (12%)	671 (12%)	4700 (11%)
Dietary intake (unit), median (p10, p90)					
Total energy (kcal/d)	2842 (1771, 4279)	2618 (1728, 3770)	2498 (1699, 3499)	2424 (1703, 3345)	2585 (1724, 3760)
Fruits, berries, and vegetables (g/d)	200 (65, 470)	230 (94, 480)	270 (120, 510)	330 (170, 570)	250 (99, 510)
Whole grains (g/d)	44 (0, 330)	100 (0, 320)	130 (35, 280)	130 (59, 230)	110 (0, 310)
Pulses (g/d)	22 (0, 72)	22 (0, 77)	24 (0, 79)	40 (19, 93)	24 (0, 79)
Nuts (g/d)	0 (0, 2.5)	0 (0, 2.5)	0 (0, 2.5)	0.34 (0, 2.6)	0 (0, 2.5)
Unsaturated oil	0 (0, 0)	0 (0, 0)	0 (0, 0)	0 (0, 0)	0 (0, 0)
Total fish (g/d)	27 (7.5, 67)	31 (13, 63)	35 (17, 65)	43 (21, 68)	33 (14, 65)
Egg (g/d)	5.5 (0, 41)	15 (4.8, 36)	15 (4.8, 18)	15 (4.8, 18)	15 (4.8, 36)
Potatoes (g/d)	150 (50, 230)	130 (53, 190)	100 (53, 180)	100 (53, 150)	120 (53, 190)
White meat (g/d)	7.8 (0, 29)	8.9 (0, 25)	8.9 (0, 25)	8.9 (0, 29)	8.9 (0, 25)
Juice (g/d)	14 (0, 210)	14 (0, 160)	14 (0, 100)	14 (0, 100)	14 (0, 150)
Dairy products (g/d)	440 (0, 1200)	430 (62, 1000)	420 (140, 840)	400 (210, 670)	420 (66, 980)
Red meat (g/d)	80 (25, 130)	60 (23, 89)	48 (22, 83)	45 (17, 70)	55 (22, 88)
Processed meat (g/d)	40 (19, 87)	38 (15, 73)	34 (10, 66)	24 (5.2, 60)	36 (10, 72)
Added sugar (g/d)	10 (10, 17)	10 (10, 14)	10 (10, 12)	10 (10, 11)	10 (10, 14)
Caffeine	390 (140, 800)	330 (130, 570)	290 (120, 500)	270 (120, 440)	310 (130, 570)

Abbreviations: *N,* sample size; NNR23, Nordic Nutrition Recommendations 2023.

**FIGURE 1:**
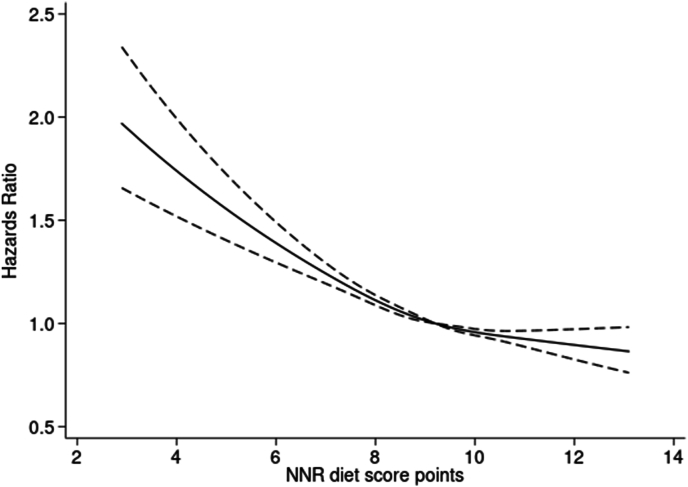
Adherence to Nordic Nutrition Recommendations (NNR) 2023 and risk of all-cause mortality modeled with a restricted cubic spline. Model 3: age (y), sex (men/women), energy (kcal/d). Education (primary, high school, university), income (SEK in quintiles, multiplied by 100), sleep (h/d; < 6, 6-<7, 7–<8, 8-<9, >9), smoking status (never, former smokers (<20, 20–<40, or >40 cigarettes/d), or current smokers (<20, 20–39, or >40 cigarettes/d), alcohol (g/d), walking/cycling (min/a day, <20, 20–40, 40–60, or >60), dietary supplements (regularly, sometimes, no)^,^ BMI [underweight (BMI <18.5), normal weight (BMI 18.5–<2524.9), overweight (BMI 25–<30), obese (BMI >30)], hypertension (yes, no), diabetes (yes, no), and hypercholesterolemia (yes, no). The analysis was conducted using Cox proportional hazards model. Nonlinearity; *P* = 0.0003.

During a median follow-up time of 18.8 y (6,049,513 person-years), 30,142 participants died (17,121 men and 13,021 women). Participants with the highest adherence (>10 points) to NNR23 at baseline had lower all-cause mortality compared with participants with the lowest adherence (<8 points) (HR 0.77; 95% CI: 0.74, 0.80), model 3, Table 3). For the analysis using long-term average intake, participants with the highest adherence also had lower all-cause mortality compared with participants with the lowest adherence [>10 points compared with <8 points, HR 0.38 (95% CI: 0.37, 0.40), model 3, Table 3]. When assessing NNR23 score as a restricted cubic spline with 4 knots, the analysis showed a nonlinear association for lower mortality with higher adherence to NNR23 (*P* = 0.0003) (Figure 1).

**TABLE 3:** Adherence to Nordic Nutrition Recommendations 2023 at baseline 1997 and long-term (1997, 2009, and 2019) and risk of all-cause mortality among 76,122 men and women from the Cohort of Swedish Men and the Swedish Mammography Cohort.

	NNR23 diet score in points				*P*-trend
	0–8 points	>8–9 points	>9–10 points	>10–13 points	
Baseline intake, 1997					
	(*N =* 11,226)	(*N =* 20,958)	(*N =* 27,594)	(*N =* 16,344)	

Cases, *n*	4434	8426	10,890	6392	
Person-years	864,006	1,651,811	2,209,651	1,324,046	
HR 95% CI					
Model 1^1^	Ref.	0.82 (0.79, 0.85)	0.73 (0.70, 0.76)	0.68 (0.65, 0.70)	<0.0001
Model 2^2^	Ref.	0.88 (0.84, 0.91)	0.81 (0.78, 0.84)	0.78 (0.75, 0.81)	<0.0001
Model 3^3^	Ref.	0.88 (0.84, 0.91)	0.81 (0.78, 0.84)	0.77 (0.74, 0.80)	<0.0001

Long-term average intake, 1997, 2009, and 2019					
	*N* = 5074	*N* = 11,320	*N =* 21,584	*N =* 38,144	*P*-trend

Cases, *n*	3181	6495	10,107	10,359	
Person-years	389,248	892,094	1,721,832	3,046,338	
HR 95% CI					
Model 1^1^	Ref.	0.73 (0.70, 0.77)	0.56 (0.53, 0.58)	0.34 (0.33, 0.36)	<0.0001
Model 2^2^	Ref.	0.76 (0.73, 0.80)	0.60 (0.57, 0.62)	0.38 (0.37, 0.40)	<0.0001
Model 3^3^	Ref.	0.76 (0.72, 0.79)	0.60 (0.57, 0.63)	0.38 (0.37, 0.40)	<0.0001

The HRs were calculated using Cox proportional hazards models.

Abbreviations: CI, confidence interval; HR, hazard ratio; NNR, Nordic Nutrition Recommendations.

1 Model 1: age (y) and sex-adjusted (men/women), energy intake (kcal/d).

2 Model 2: + education (primary, high school, university), income (quintiles, Swedish Kronor), sleep (<6, 6–<7, 7–<8, 8–<9, >9 h/d), smoking status [never, former smokers (<20, 20–<40, or >40 cigarettes/d), or current smokers (<20, 20–39, or >40 cigarettes/d)], alcohol (g/d), walking/cycling (<20, 20–40, 40–60, 60–<90, >90 min/d), dietary supplements (regularly, sometimes, no).

3 Model 3: + BMI, kg/m^2^ (<18.5 underweight, 18.5–<25 normal weight, 25–<30 overweight, >30 obesity), hypertension (yes, no), diabetes (yes, no), hypercholesterolemia (yes, no).

In analyses with cause-specific mortality, we found that cardiovascular mortality showed a similar pattern to the main all-cause mortality analysis, with a clear and strong inverse association with higher adherence to the NNR23 diet (>10 points compared with <8 points HR 0.77; 95% CI: 0.72, 0.82). The association with cancer mortality (>10 points compared with <8 points HR 0.83; 95% CI: 0.77, 0.89) was slightly weaker and appeared to plateau at moderate adherence levels (Table 4).

**TABLE 4:** Adherence to Nordic Nutrition Recommendations 2023 at baseline 1997 and risk of cancer- and cardiovascular mortality among men and women from the Cohort of Swedish Men and the Swedish Mammography Cohort.

	NNR diet score in points			
	0–8 points	>8–9 points	>9–10 points	>10–13 points
Cancer mortality				
	(*N =* 11,226)	(*N =* 20,958)	(*N =* 27,594)	(*N =* 16,344)

Cases, *n*	1678	3015	3859	2271
HR 95% CI				
Model 3^1^	Ref.	0.89 (0.83, 0.94)	0.83 (0.78, 0.89)	0.83 (0.77, 0.89)

Cardiovascular mortality				
	(*N =* 11,226)	(*N =* 20,958)	(*N =* 27,594)	(*N =* 16,344)

Cases, *n*	2064	4066	5502	3211
HR 95% CI				
Model 3^1^	Ref.	0.87 (0.82, 0.92)	0.83 (0.78, 0.87)	0.77 (0.72, 0.82)

The HRs were calculated using Cox proportional hazards models.

Abbreviations: CI, confidence interval; HR, hazard ratio; NNR, Nordic Nutrition Recommendations.

1 Model 3: age (y) and sex-adjusted (men/women), energy (kcal/d), education (primary, high school, university), income (quintiles, Swedish Kronor), sleep (<6, 6–<7, 7–<8, 8–<9, >9 h/d), smoking status [never, former smokers (<20, 20–<40, or >40 cigarettes/d), or current smokers (<20, 20–39, or >40 cigarettes/d], alcohol (g/d), walking/cycling (<20, 20–40, 40–60, 60–<90, >90 min/d), dietary supplements (regularly, sometimes, no) energy intake (kcal/d), BMI, kg/m^2^ (<18.5 underweight, 18.5–<25 normal weight, 25–<30 overweight, >30 obesity), hypertension (yes, no), diabetes (yes, no), hypercholesterolemia (yes, no).

### Analysis stratified by sex, BMI, diabetes, and income

We observed similar results for men and women when analyzing their data separately (Supplemental Table 3). Therefore, we presented the main results for the 2 cohorts combined.

Stratifying the analysis on BMI showed that participants with normal weight, overweight, or obesity had similar lower mortality with higher adherence to NNR23 as the main analysis (Supplemental Table 3). When comparing the highest and lowest adherence groups, the greatest reduction in mortality was observed among underweight participants. However, this group had fewer cases (*n =* 728) compared with the other BMI categories. (Supplemental Table 3; HR 0.59; 95 % CI: 0.41, 0.83). We observed a stronger inverse association between adherence to the diet score and mortality among participants without diabetes at baseline (HR 0.58; 95% CI: 0.41, 0.82; Supplemental Table 3), compared with those with diabetes (HR: 0.78; 95% CI: 0.75, 0.82). When stratified by income, we observed an inverse association between adherence to the diet score and mortality across all income groups, ranging from 0.75 (95% CI: 0.69; 0.82; Supplemental Table 3) in the lowest income group to 0.84 (95% CI: 0.75, 0.94) in the highest. We did not find any significant interactions for either sex, BMI, diabetes, or income (Supplemental Table 3).

### Sensitivity analysis

In the sensitivity analysis based on the baseline diet, we removed 1 of 15 food components at a time from the total NNR23 score. Of those 15 separate analyses based on a limited NNR23 score, 12 showed weaker associations than the main analysis. The 3 exceptions, for which results were very similar (∼22% lower mortality) as for the total NNR23 score in the main analysis, were observed for the limited NNR23 scores without pulses, without nuts and seeds, or without unsaturated oils (Supplemental Figure 3, Supplemental Table 4).

When excluding the first year of mortality, the association was similar to the main analyses [>10 points compared with <8 points participants in higher adherence categories had progressively lower mortality risk, with HRs of 0.88 (95% CI: 0.85, 0.92), HR 0.80 (95% CI: 0.77, 0.84), and HR 0.75 (95% CI: 0.72, 0.78), compared with the lowest adherence group; Supplemental Table 5]**.**

When restricting the study population to participants with different combinations of repeat dietary data, we observed broadly similar directions of associations as in the main results using baseline dietary data only, albeit with very wide CIs due to the low number of deaths among those who survived until 2019 (Supplemental Table 6).

## Discussion

In this study, we developed a novel food-based diet score representing adherence to NNR23 dietary guidelines. We observed a 23% lower risk of all-cause mortality when comparing highest to lowest adherence to NNR2023 at baseline among Swedish middle-aged and elderly adults. When including data on food intake from 2009 and 2019, we saw a 62% lower risk of all-cause mortality when comparing the highest to the lowest adherence to NNR2023. Results were similar when assessing cardiovascular mortality and cancer mortality.

### The food-based NNR23 score

The updated NNR from 2023 introduced food-based guidelines, whereas the previous recommendations from 2012 were primarily nutrient-based [6,22]. For the novel food-based NNR23 diet score, we used a proportional scoring system ranging from 0 to 1 on a continuous scale to better capture the nuances in participants' dietary intake. Compared with other dietary guidelines with a planetary focus such as the EAT-Lancet [5], NNR is less strict in its recommendations and does not necessarily quantify all its recommendations, reflecting a more pragmatic approach, which may have contributed to the observed minimal variation in NNR23 diet score, with most of the participants scoring over 8 points.

One limitation of the score in our study is the assumption that food intake estimated from FFQs captures absolute values of consumption. Although studies have shown that both under- and overreporting of food intakes may take place when using FFQs in cohort studies [[24], [25], [26], [27]], they are considered useful for gathering diet information and rank-ordering participants. Our scoring method assigns points on a continuous scale, so, for example, overreporting vegetable intake would lead to only a slightly higher score than the true intake. Therefore, this method is assumed to be superior to the most often used binary scoring system based on strict cut-offs, where misclassification between only 2 scoring categories (0 or 1 score) can easily occur for self-reported intakes close to cutoff points. Assessing the effects of adherence to some items, such as nuts and seeds, and pulses, was challenging because these foods were not commonly consumed in Sweden in the 1990s [23]. Evaluation of adherence to recommendations regarding unsaturated oils was also difficult because the available data were limited. Recommendations for pulses, egg, potatoes, juice, and added sugar were not quantified, and in such cases, we used reference intake levels within the individual food chapters as a proxy for recommended intake. However, average intake does not necessarily represent a safe limit or ensure optimal health benefits [6]. To align with NNR23 emphasis on balanced nutritional and energy intake, we set an upper limit for all food components. This approach, however, effectively penalized participants who consumed high amounts of beneficial foods, albeit very few participants exceeded these thresholds for beneficial food groups.

To the best of our knowledge, only 1 other diet score assesses adherence to NNR23 in relation to a health outcome [7]. This score includes 8 groups of micro- and macronutrients and physical activity and is thus very different from the food-based score we present here. In that study, using nutrient-based NNR23 participants with the highest adherence had a 28% lower risk of myocardial infarction (HR 0.72; 95% CI: 0.59, 0.87) compared with the low adherence group [7]. Recently, a study assessing the performance of 7 diet scores based on the EAT-Lancet reference diet highlighted the variability in diet scores derived from the same recommendations. Generally, scores awarding points on a continuous scale relative to participants’ intakes performed better than those awarding points only when the recommended level was achieved (i.e., binary scores) [28]. This was also recently observed in a study of the Planetary Health Diet and mortality, which additionally also compared single dietary measures to updated dietary data across follow-up, finding that cumulatively updated dietary data were associated with lower mortality risk [29].

### Adherence to NNR23 and mortality

Potential mechanisms for lowering mortality that are supported by a high adherence to NNR23 include greater dietary fiber intake from whole grains, vegetables, and fruits, which positively affects digestive health and short-chain fatty acid metabolism [30,31]. Furthermore, nuts, which are rich in dietary fiber, vitamins, minerals, phytosterols, and phenolic compounds, have been linked to a lower risk of several morbidities in which oxidative stress plays a key role [[32], [33], [34]]. Fish, particularly fatty fish, is another important component due to the high ω-3 fatty acid content, which has been significantly associated with a lower risk of coronary events and overall mortality [35].

In the analysis using long-term intake, we observed a greater reduction in mortality and a stronger association than in the main analysis. Selection bias could play a role, as participants who survived and responded to the next data collection may represent a healthier subset of the population. Our sensitivity analyses among those with 2 or 3 repeated dietary assessments showed associations in a similar direction as the long-term intake analysis, although, where data from 2019 were included, wide CIs were observed. Hence, neither refuting nor supporting this argument. Alternatively, the stronger association may also be explained by a broad improvement in dietary quality, as the proportion of participants in the highest adherence category is greater when using average diet during follow-up than the remaining participants in the reference group with a lower average diet score.

When stratifying by BMI groups, we observed a greater reduction in mortality in the underweight group. However, this group was very small, with only 728 participants of the total 76,122, which is reflected in the wide CI. When stratifying by diabetes status, we observed a slightly stronger inverse association between adherence to the diet score and all-cause mortality among participants without diabetes at baseline, compared with those with diabetes, suggesting that the potential protective effect of the diet may be more pronounced in individuals without pre-existing diabetes. An inverse association was also observed across all income groups, although slightly attenuated among those with higher income.

Sensitivity analyses excluding early deaths supported the robustness of the findings, with similar results as the main analysis.

In the analysis where individual items were removed from the score, all exclusions except for pulses, nuts, and seeds, and unsaturated oils resulted in HRs between 0.80 and 0.84—higher than the main analysis (0.77 HR). Removing these items from the score resulted in HRs slightly closer to 1, suggesting that intake of these foods is important for the association with mortality. However, our results do not exclude that pulses, nuts and seeds, and unsaturated oils are also important. As these foods were less commonly consumed in our study population, few participants scored highly on these components, reducing the power to investigate their contribution to the association with mortality.

A strength of this study is its prospective, population-based design with a large sample size, complete follow-up, and availability of a broad spectrum of potential confounders. Moreover, repeated collection of dietary data allows for long-term assessments of diet. However, dietary intake was measured using self-reported FFQ, and although the FFQ has shown moderate-to-strong validity [12,13], we cannot rule out the possibility of measurement error. Due to the prospective design, the measurement error is unlikely to be differential with regard to mortality and would therefore result in bias toward no association. Despite comprehensive adjustment for potential confounders, including several lifestyle and sociodemographic factors and even family income from register information, residual and unmeasured confounding cannot be completely ruled out. For example, our measure of physical activity only accounts for walking and cycling, and leisure time activities were thus unmeasured. Participants in this study are considered comparable with the general Swedish population with regards to education, and BMI [12], as well as food intake [23]. Thus, the observed association between adherence to the NNR23 score and lower mortality is likely to hold more widely for populations of similar ages, lifestyles, and socioeconomic backgrounds.

In conclusion, using a newly developed food-based diet score reflecting NNR23 adherence, we found that higher adherence was associated with lower mortality in a Swedish population.

## Author contributions

The authors’ responsibilities were as follows – AW: conducted research (data collection) and provided essential databases (necessary for the research); ABM, DBI: performed the statistical analysis; AMB: wrote the first draft of the article; CCD: primarily responsible for the final content; and all authors: designed the research (project conception, development of overall research plan, and study oversight), critically revised the text and approved the final version.

## Data availability

Data described in the manuscript, code book, and analytic code will be made available upon request through the Swedish National Research Infrastructure, SIMPLER (www.simpler4health.se).

## Funding

This study was funded by a full PhD stipend from Aarhus University Graduate School of Health, and the research grant no. 2024-0966 from Formas within the Swedish Research Program on Climate. The database used in the study is from SIMPLER (Swedish Infrastructure for Medical Population-based Life-course and Environmental Research) funded by the Swedish Research Council under grant nos. 2017-00644 and 2021-00160.

## Conflict of interest

The authors have nothing to declare.
