# Supplementary material for: Development of the Nordic Nutrition Recommendations 2023 Food-Based Diet Score and Its Association with All-Cause Mortality in Two Swedish Cohorts
Source: J Nutr. 2025 Jul 2;155(11):3747–56. doi: 10.1016/j.tjnut.2025.06.030 (PMC12799448; doi:10.1016/j.tjnut.2025.06.030)
Supplement: Multimedia component 1 [file mmc1.docx]

**Development of the Nordic Nutrition Recommendations 2023 food-based diet score and its association with all-cause mortality in two Swedish cohorts**

Anne B. Mørch^1,4^, Daniel B. Ibsen^1,2,3^, Alicja Wolk^4^ and Christina C. Dahm^1^

^1^Department of Public Health, Aarhus University, Aarhus, Denmark; ^2^Steno Diabetes Center Aarhus, Aarhus, Denmark; ^3^Department of Nutrition, Exercise and Sports, University of Copenhagen, Copenhagen, Denmark; ^4^Department of Environmental Medicine, Karolinska Institutet, Stockholm, Sweden.

**Corresponding author**: Christina C. Dahm; Department of Public Health, Aarhus University, Denmark; Bartholins Allé 2, DK-8000 Aarhus C, Denmark. E-mail: [ccd@ph.au.dk](mailto:ccd@ph.au.dk)

**Supplementary material**

| **Supplementary Table 1.** Documentation of the development of Nordic Nutrition Recommendations 2023 diet score. | | | | | | |
| --- | --- | --- | --- | --- | --- | --- |
|  | **Points** | | | **Component type** | **Notes** | |
| **Index component** | 0 | 1 | 0 |  | |  |
|  |  |  |  |  | |  |
| Vegetables, fruit, and berries | 0 g/day | 500-800 g/day | >1,600 g/day | Encourage | | Specified range from NNR23, Table 24. |
| Wholegrain Cereal | 0 g/day | 90-210 g/day | >420 g/day | Encourage | | NNR23, Table 24 recommends at least 90 g/day with likely benefits with higher intakes. In a previous iteration of NNR23 they specify this “higher intake” to 210 g/day, which we have chosen to adopt. |
| Pulses | 0 g/day | 100 g/day | >200 g/day | Encourage | | NNR23 does not quantify this recommendation but refers to a study with shown health benefits up to 100 g/day. |
| Nuts and seeds | 0 g/day | 20-30 g/day | >60 g/day | Encourage | | Specified range in NNR23, Table 24. |
| Unsaturated oils | 0 g/day | 25 g/day | >50 g/day | Encourage | | Specified range in NNR23, Table 24. |
| Fish and seafood | 0 g/day | 43-64 g/day including 28.5 g/day fatty fish | >128 g/day including >57 g/day fatty fish | Encourage | | Specified range in NNR23, Table 24. |
| Egg |  | 0-60 g/day | >120 g/day | Moderate | | NNR23 does not quantify this recommendation, but states that average intake is about 10-40 g/day. In this case, we have chosen to set the limit to 0-60 g/day for practical reasons, as 60 grams is the average weight for 1 egg. |
| Potatoes | 0 g/day | 50-130 g/day | >260 g/day | Moderate | | NNR23 does not quantify the recommendation on potatoes but within the chapter on potatoes, they refer to positive health effects at intake in the range of 50-130 g/day. We have chosen to adopt this range into the diet score. |
| White meat |  | 0-50g/day | >100 g/day | Moderate | | NNR23, Table 24, states intake should be as low as possible. Average intake in the Nordic and Balic countries is 20-50 g/day and intake should not increase. We have chosen to adopt that range. |
| Juice |  | 0-100 g/day | >200 g/day | Moderate | | NNR23 does not quantify this recommendation and does not specify an average intake, so we have set this guideline to 100 g/day equivalent to 1 glass pr. Day. |
| Milk and dairy | 0 g/day | 250-500 g/day | >1000 g/day | Moderate | | Specified range in NNR23, Table 24. |
| Red meat |  | 0-50 g/day | >100 g/day | Discourage | | Specified range from NNR23, Table 24. |
| Processed meat |  | 0-10 | >20 | Discourage | | In NNR23, Table 24 recommends as little as possible should be consumed. Few individuals will have an intake of 0 g/day making it a redundant component. Therefore, we chose 0-10 g/day as the appropriate range. Also, FFQs can attribute intake of a food from standard recipes that were not consumed by an individual. |
| Added sugar |  | <10E%/day | >20E%/day | Discourage | | NNR23 does not specify a range but have with the earlier 2012 version established that added sugar should be kept under 10 E%. We have chosen to adopt this limit. |
| Caffeine from coffee and tea |  | 0-400 mg/day | >800 mg/day | Moderate | | In NNR23 chapter on beverages, the recommended intake range for caffeine is 0-400 mg of caffeine a day. |

NNR23, Nordic Nutrition Recommendations 2023.

| **Supplementary Table 2**. Development of the diet score based on the Nordic Nutrition Recommendations 2023. | | | | | |
| --- | --- | --- | --- | --- | --- |
|  |  | **Points** | | | **Full adherence, n (%)** |
|  | **Score components** | 0 | 1 (recommended range) | 0 |  |
| **Association with health** |  |  |  |  |  |
|  |  |  |  |  |  |
| **Encourage** | Vegetables, fruit, and berries | 0 g/day | 500-800 g/day | >1600 g/day | 5,988 (7.86 %) |
|  | Wholegrain Cereal | 0 g/day | 90-210 g/day | >420 g/day | 23,050 (30.28 %) |
|  | Pulses | 0 g/day | 100 g/day | >200 g/day | 0 (0 %) |
|  | Nuts and seeds | 0 g/day | 20-30 g/day | >60 g/day | 146 (0.19 %) |
|  | Unsaturated oils | 0 g/day | 25 g/day | >50 g/day | 0 (0 %) |
|  | Fish and seafood | 0 g/day | 43-64 g/day including 28,5 g/day fatty fish | >128 g/day including >57 g/day fatty fish | 1,725 (2.26 %) |
|  |  |  |  |  |  |
| **Moderate** | Egg |  | 0-60 g/day | >120 g/day | 74,678 (98.10 %) |
|  | Potatoes | 0 g/day | 50-130 g/day | >260 g/day | 46,789 (61.46 %) |
|  | White meat |  | 0-50 g/day | 100 g/day | 75,230 (98.82 %) |
|  | Juice |  | 0-100 g/day | >200 g/day | 64,395 (84.59 %) |
|  | Milk and dairy | 0 g/day | 350-500 g/day | >1000 g/day | 14,710 (19.32 %) |
|  |  |  |  |  |  |
| **Discourage** | Red meat |  | 0-50 g/day | >100 g/day | 49,107 (64.51 %) |
|  | Processed meat |  | 0-10 g/day | >20 g/day | 9,701 (12.74 %) |
|  | Added sugar |  | <10E% pr. day | >20E% pr. day | 56,847 (74.67 %) |
|  | Caffeine from coffee and tea |  | 0-400 mg/day | >800 mg | 58,117 (76,34 %) |

| **Supplementary Table 3**. Association between adherence to the Nordic Nutrition Recommendations 2023 diet score at baseline and the risk of all-cause mortality stratified on sex, BMI, diabetes and income. | | | |  |
| --- | --- | --- | --- | --- |
|  | **Cases, n** | **HR (95% CI)** |  | **Interaction** |
| **Sex**^1^ |  | **0-8 points** | **>10-13 points** | p= 0.06 |
| Men, N= 41,118 | 17,101 | Ref. | 0.81 (0.76,0.86) |  |
| Women, N= 35,004 | 13,021 | Ref. | 0.78 (0.73,0.84) |  |
| **BMI**^2^ |  |  |  | p=0.23 |
| Underweight, N = 728 | 443 | Ref. | 0.59 (0.41,0.83) |  |
| Normal weight, N = 36,112 | 13,272 | Ref. | 0.79 (0.78,0.84) |  |
| Overweight, N = 29,306 | 11,640 | Ref. | 0.77 (0.72,0.82) |  |
| Obese, N = 7,345 | 3,266 | Ref. | 0.75 (0.66,0.85) |  |
| **Diabetes**^3^ |  |  |  | p=0.09 |
| No, N = 72,535 | 27717 | Ref. | 0.58 (0.41,0.82) |  |
| Yes, N = 3,587 | 2425 | Ref. | 0.78 (0.75,0.82) |  |
| **Income**^4^**, (SEK in quintiles)** |  |  |  | p=0.78 |
| < 88,400, N= 15,266 | 8584 | Ref. | 0.75 (0.69,0.82) |  |
| 88,400 –114,000, N= 15,189 | 8158 | Ref. | 0.77 (0.71,0.84) |  |
| 114,000 – 141,000, N= 15,226 | 5584 | Ref. | 0.80 (0.72,0.84) |  |
| 141,000 – 176,000, N= 15,204 | 4129 | Ref. | 0.84 (0.75,0.94) |  |
| >176,000, N= 15,207 | 3681 | Ref. | 0.80 (0.71,0.90) |  |
| N= sample size; HR, hazards ratio; CI, confidence interval; BMI, body mass index. The hazard ratio was calculated using Cox proportional hazards models.  ^1^Model3: age (years), energy (kcal per day, education (primary, high school, university), income (SEK in quintiles, multiplied by 100), sleep (hours pr. day; < 6, 6-<7, 7-<8, 8-<9, >9), smoking status (never, former smokers (<20, 20-<40, or >40 cigarettes pr. day), or current smokers (<20, 20–39, or >40 cigarettes pr. day), alcohol (g/day), walking/cycling (min/a day, <20, 20–40, 40–60, 60-<90, >90), dietary supplements (regularly, sometimes, no)^,^ BMI (underweight (BMI <18.5), normal weight (BMI 18.5 <25-24.9), overweight (BMI 25-<30), obese (BMI >30)), hypertension (yes, no), diabetes (yes, no), and hypercholesterolemia (yes, no).  ^2^Model3: age (years), sex (men/women), energy (kcal per day), education (primary, high school, university), income (SEK in quintiles, multiplied by 100), sleep (hours pr. day; < 6, 6-<7, 7-<8, 8-<9, >9), smoking status (never, former smokers (<20, 20-<40, or >40 cigarettes pr. day), or current smokers (<20, 20–39, or >40 cigarettes pr. day), alcohol (g/day), walking/cycling (min/a day, <20, 20–40, 40–60, 60-<90, >90), dietary supplements (regularly, sometimes, no), hypertension (yes, no), diabetes (yes, no), and hypercholesterolemia (yes, no).  ^3^Model3: age (years), sex (men/women), energy (kcal per day), education (primary, high school, university), income (SEK in quintiles, multiplied by 100), sleep (hours pr. day; < 6, 6-<7, 7-<8, 8-<9, >9), smoking status (never, former smokers (<20, 20-<40, or >40 cigarettes pr. day), or current smokers (<20, 20–39, or >40 cigarettes pr. day), alcohol (g/day), walking/cycling (min/a day, <20, 20–40, 40–60, 60-<90, >90), dietary supplements (regularly, sometimes, no)^,^ BMI (underweight (BMI <18.5), normal weight (BMI 18.5 <25-24.9), overweight (BMI 25-<30), obese (BMI >30)), hypertension (yes, no), and hypercholesterolemia (yes, no).  ^4^Model3: age (years), sex (men, women) energy (kcal per day). education (primary, high school, university), sleep (hours pr. day; < 6, 6-<7, 7-<8, 8-<9, >9), smoking stauts (never, former smokers (<20, 20-<40, or >40 cigarettes pr. day), or current smokers (<20, 20–39, or >40 cigarettes pr. day), alcohol (g/day), walking/cycling (min/a day, <20, 20–40, 40–60, 60-<90, >90), dietary supplements (regularly, sometimes, no)^,^ BMI (underweight (BMI <18.5), normal weight (BMI 18.5 <25-24.9), overweight (BMI 25-<30), obese (BMI >30)), hypertension (yes, no), diabetes (yes, no), and hypercholesterolemia (yes, no). | | | |  |

| **Supplementary Table 4.** Association between adherence to the Nordic Nutrition Recommendation 2023 diet score at baseline and the risk of all-cause mortality excluding one food component at the time. | | |
| --- | --- | --- |
| **Adherence to NNR23 at baseline** | | |
| **Component** excluded^1^ | **0-8 points** | **>10-13 points** |
| Without red meat | Ref. | HR (95% CI)  0.84 (0.79,0.90) |
| Without processed meat | Ref. | 0.80 (0.77,0.83) |
| Without white meat | Ref. | 0.84 (0.78,0.89) |
| Without fish | Ref. | 0.80 (0.76,0.84) |
| Without egg | Ref. | 0.83 (0.78,0.89) |
| Without wholegrain | Ref. | 0.80 (0.76,0.86) |
| Without vegetables, fruit, and berries | Ref. | 0.82 (0.78,0.86) |
| Without pulses | Ref. | 0.77 (0.73,0.80) |
| Without juice | Ref. | 0.83 (0.78,0.88) |
| Without potatoes | Ref. | 0.82 (0.77,0.87) |
| Without added sugar | Ref. | 0.85 (0.80,0.91) |
| Without nuts and seeds | Ref. | 0.78 (0.75,0.82) |
| Without unsaturated oil | Ref. | 0.78 (0.75,0.81) |
| Without dairy | Ref. | 0.83 (0.79,0.88) |
| Without caffeine | Ref. | 0.85 (0.79,0.91) |
| NNR23, Nordic Nutrition Recommendations; HR, hazard ratios; CI, confidence interval. The hazard ratio was calculated using Cox proportional hazards models.  ^1^Model3: age (years), sex (men/women), energy (kcal/day), education (primary, high school, university), income (SEK in quintiles, multiplied by 100), sleep (hours pr. day; < 6, 6-<7, 7-<8, 8-<9, >9), smoking status (never, former smokers (<20, 20-<40, or >40 cigarettes pr. day), or current smokers (<20, 20–39, or >40 cigarettes pr. day), alcohol (g/day), walking/cycling (min/a day, <20, 20–40, 40–60, 60-<90, >90), dietary supplements (regularly, sometimes, no)^,^ BMI (underweight (BMI <18.5), normal weight (BMI 18.6-<25), overweight (BMI 25-<30), obese (BMI >30), hypertension (yes, no), diabetes (yes, no), and hypercholesterolemia (yes, no). | | |

| **Supplementary Table 5.** Adherence to Nordic Nutrition Recommendation 2023 at baseline (1997) and risk of all-cause mortality excluding first year mortality. | | | | |
| --- | --- | --- | --- | --- |
|  | **NNR diet score in points** | | | |
|  | **0-8 points** | **>8-9 points** | **>9-10 points** | **>10-13 points** |
| **Baseline intake, 1997** | | | | |
|  | **(N=11,145)** | **(N=20,851)** | **(N=27,482)** | **(N=16,277)** |
| **Cases, n** | **4353** | **8319** | **10778** | **6325** |
|  |  |  |  |  |
| **HR 95% CI** |  |  |  |  |
| Model 3^1^ | Ref. | \| 0.88 \| (0.85, \| 0.92) \| \| --- \| --- \| --- \| | \| 0.80 \| (0.77, \| 0.84) \| \| --- \| --- \| --- \| | \| 0.75 \| (0.72 \| 0.78) \| \| --- \| --- \| --- \| |
| NNR, Nordic Nutrition Recommendations; N= sample size; HR, hazards ratio; CI, confidence interval. The hazard ratios were calculated using Cox proportional hazards models.  ^1^Model 3: age (years) and sex-adjusted (men/women), energy intake (kcal/day), education (primary, high school, university), income (quintiles, SEK), sleep (< 6, 6-<7, 7-<8, 8-<9, >9 hours/day), smoking status (never, former smokers (<20, 20-<40, or >40 cigarettes pr. day), or current smokers (<20, 20–39, or >40 cigarettes pr. day), alcohol (g/day), walking/cycling (<20, 20–40, 40–60, 60-<90, >90 min/day), dietary supplements (regularly, sometimes, no)^,^ , kg/m^2^ (<18.5 underweight, 18.5-<25 normal weight, 25-<30 overweight, >30 obesity), hypertension (yes, no), diabetes (yes, no), hypercholesterolemia (yes, no). | | | | |

| **Supplementary Table 6.** Adherence to Nordic Nutrition Recommendations 2023 in 1997, 2009, and 2019 risk of all-cause mortality among participant with data from either two or three time points. | | | | |
| --- | --- | --- | --- | --- |
|  | **NNR23 diet score in points** | | | |
|  | **0-8 points** | **>8-9 points** | **>9-10 points** | **>10-13 points** |
| **Two time points (1997 and 2009)** | | | | |
|  | **(N= 6,302)** | **(N= 12,095)** | **(N= 16,525)** | **(N= 9,963)** |
| **Cases, n** | 1,486 | 2,922 | 3,929 | 2,354 |
|  |  |  |  |  |
| **HR 95% CI** |  |  |  |  |
| Model 3^1^ | Ref. | 0.88 (0.82,0.94) | 0.80 (0.75,0.85) | 0.76 (0.71,0.82) |
| **Two time points (1997 and 2019)** | | | | |
|  | **(N= 4,062)** | **(N= 7,605)** | **(N= 10,147)** | **(N= 6,065)** |
| **Cases, n** | 8 | 20 | 30 | 13 |
|  |  |  |  |  |
| **HR 95% CI** |  |  |  |  |
| Model 3^1^ | Ref. | 1.20 (0.52, 2.78) | 1.36 (0.60, 3.10) | 0.86 (0.33, 2.22) |
| **Three time points (1997, 2009, and 2019)** | | | | |
|  | **(N= 3,369)** | **(N= 6,487)** | **(N= 8,797)** | **(N= 5,355)** |
| **Cases, n** | 8 | 17 | 24 | 13 |
| **HR 95% CI** |  |  |  |  |
| Model 3^1^ | Ref. | 0.99 (0.42,2.35) | 0.99 (0.42, 2.34) | 0.80 (0.30, 2.10) |
| NNR23, Nordic Nutrition Recommendation 2023, N= sample size; HR, hazard ratio; CI, confidence interval. The hazard ratio was calculated using Cox proportional hazards models.  ^1^Model 3: age (years) and sex-adjusted (men/women), energy intake (kcal/day), education (primary, high school, university), income (quintiles, SEK), sleep (< 6, 6-<7, 7-<8, 8-<9, >9 hours/day), smoking status (never, former smokers (<20, 20-<40, or >40 cigarettes pr. day), or current smokers (<20, 20–39, or >40 cigarettes pr. day), alcohol (g/day), walking/cycling (<20, 20–40, 40–60, 60-<90, >90 min/day), dietary supplements (regularly, sometimes, no), BMI, kg/m^2^ (<18.5 underweight, 18.5-<25 normal weight, 25-<30 overweight, >30 obesity), hypertension (yes, no), diabetes (yes, no), hypercholesterolemia (yes, no). | | | | |

| 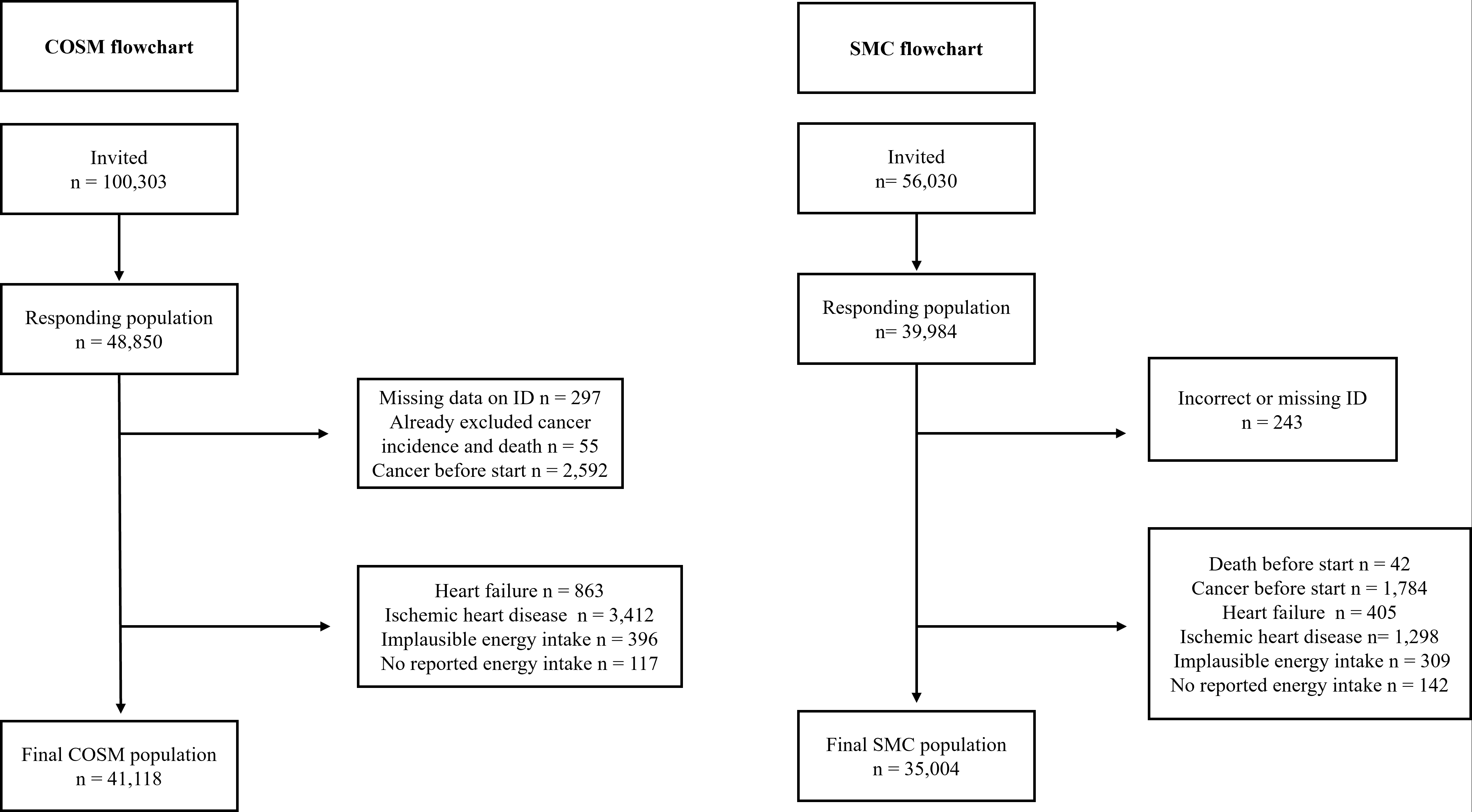 |
| --- |
| **Supplementary Figure 1**. Flowchart of in- and exclusions of the study population. |
|  |
| 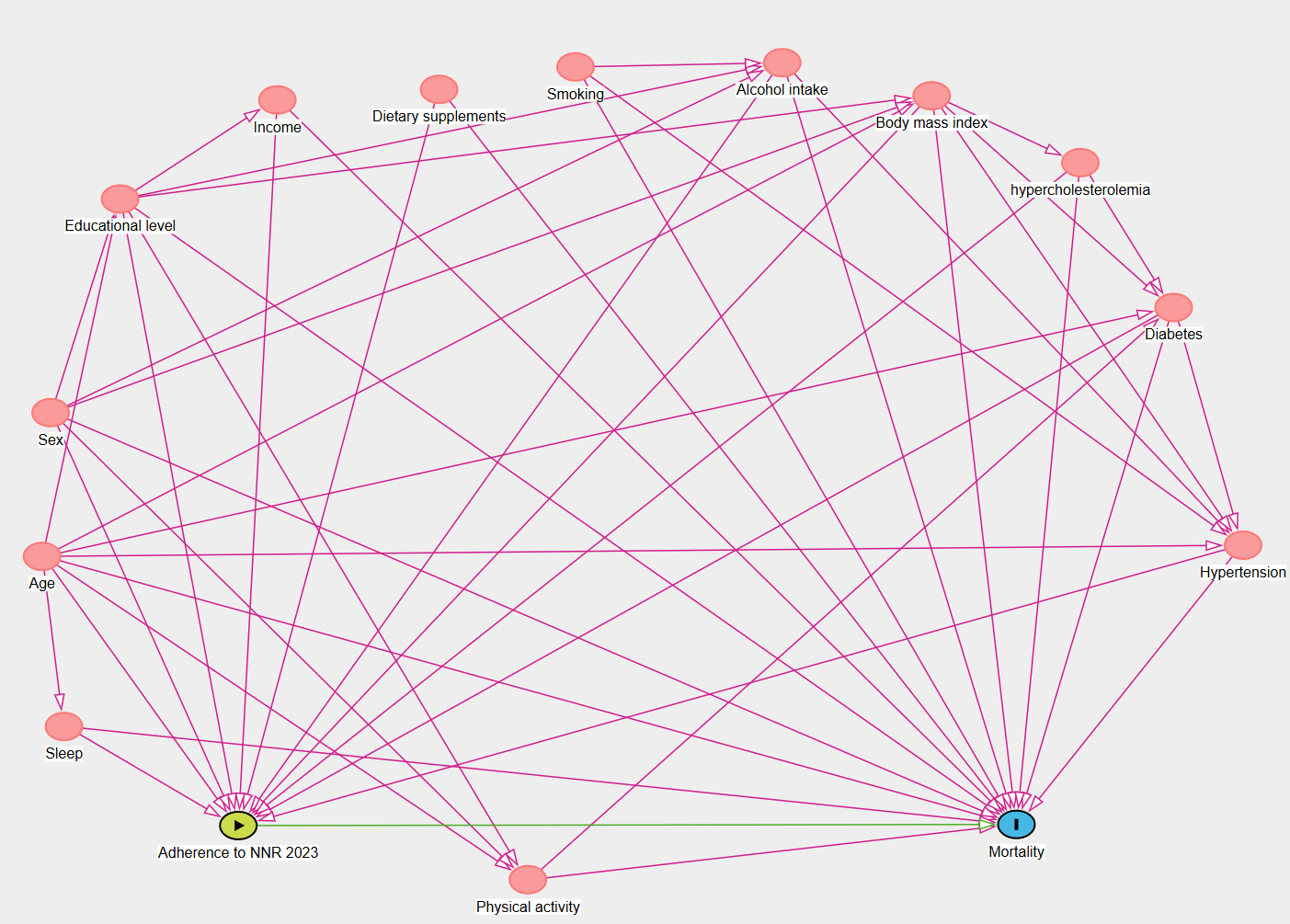  **Supplementary Figure 2:** Directed Acyclic Graph. Illustration of the proposed relationship between adherence to Nordic Nutrition Recommendation 2023, mortality, and covariates. |
| \| **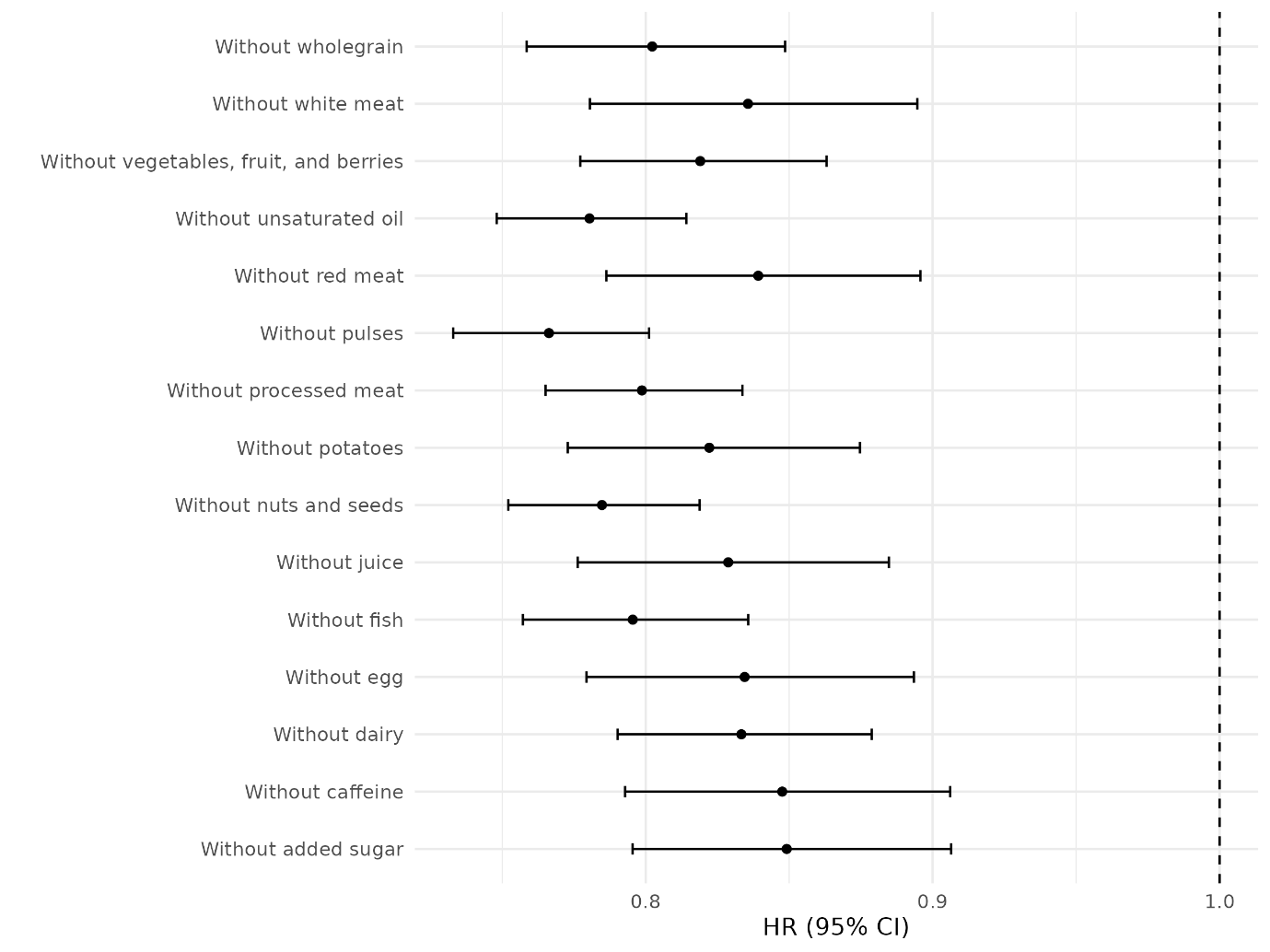** \| \| --- \| \| **Supplementary Figure 3.** Association between adherence to the Nordic Nutrition Recommendation 2023 diet score and the risk of mortality excluding one item at the time^1^.  The hazard ratios were calculated using Cox proportional hazards models.  ^1^Model3: age (years), sex (men/women), energy (kcal/day), education (primary, high school, university), income (SEK in quintiles, multiplied by 100), sleep (hours pr. day; < 6, 6-<7, 7-<8, 8-<9, >9), smoking status (never, former smokers (<20, 20-<40, or >40 cigarettes pr. day), or current smokers (<20, 20–39, or >40 cigarettes pr. day), alcohol (g/day), walking/cycling (min/a day, <20, 20–40, 40–60, 60-<90, >90), dietary supplements (regularly, sometimes, no)^,^ BMI (underweight (BMI <18.5), normal weight (BMI 18.5-<25), overweight (BMI 25-<30), obese (BMI >30)), hypertension (yes, no), diabetes (yes, no), and hypercholesterolemia (yes, no). \| |
